# Prognosis signature for predicting the survival and immunotherapy response in esophageal carcinoma based on cellular senescence-related genes

**DOI:** 10.3389/fonc.2023.1203351

**Published:** 2023-08-17

**Authors:** Yue Wang, Longfei Dai, Ran Huang, Weisong Li, Wenyong Wu

**Affiliations:** ^1^ Anhui No.2 Provinicial People's Hospital Clinical College of Anhui Medical University, Hefei, China; ^2^ Department of General Surgery, Anhui No.2 Provinicial People's Hospital, Hefei, China; ^3^ The Fifth Clinical Medical College of Anhui Medical University, Hefei, China; ^4^ Department of Pediatric Surgery, The First Affiliated Hospital of Anhui Medical University, Hefei, China; ^5^ Department of General Surgery, The First Affiliated Hospital of Anhui Medical University, Hefei, China

**Keywords:** cellular senescence, esophageal carcinoma, prognosis signature, immunotherapy, bioinformatic analysis

## Abstract

**Background:**

Cellular senescence occurs throughout life and can play beneficial roles in a variety of physiological processes, including embryonic development, tissue repair, and tumor suppression. However, the relationship between cellular senescence-related genes (CSRGs) and immunotherapy in esophageal carcinoma (ECa) remains poorly defined.

**Methods:**

The data set used in the analysis was retrieved from TCGA (Research Resource Identifier (RRID): SCR_003193), GEO (RRID: SCR_005012), and CellAge databases. Data processing, statistical analysis, and diagram formation were conducted in R software (RRID: SCR_001905) and GraphPad Prism (RRID: SCR_002798). Based on CSRGs, we used the TCGA database to construct a prognostic signature for ECa and then validated it in the GEO database. The predictive efficiency of the signature was evaluated using receiver operating characteristic (ROC) curves, Cox regression analysis, nomogram, and calibration curves. According to the median risk score derived from CSRGs, patients with ECa were divided into high- and low-risk groups. Immune infiltration and immunotherapy were also analyzed between the two risk groups. Finally, the hub genes of the differences between the two risk groups were identified by the STRING (RRID: SCR_005223) database and Cytoscape (RRID: SCR_003032) software.

**Results:**

A six-gene risk signature (DEK, RUNX1, SMARCA4, SREBF1, TERT, and TOP1) was constructed in the TCGA database. Patients in the high-risk group had a worse overall survival (OS) was disclosed by survival analysis. As expected, the signature presented equally prognostic significance in the GSE53624 cohort. Next, the Area Under ROC Curve (AUC=0.854) and multivariate Cox regression analysis (HR=3.381, 2.073-5.514, *P*<0.001) also proved that the risk signature has a high predictive ability. Furthermore, we can more accurately predict the prognosis of patients with ECa by nomogram constructed by risk score. The result of the TIDE algorithm showed that ECa patients in the high-risk group had a greater possibility of immune escape. At last, a total of ten hub genes (APOA1, MUC5AC, GC, APOA4, AMBP, FABP1, APOA2, SOX2, MUC8, MUC17) between two risk groups with the highest interaction degrees were identified. By further analysis, four hub genes (APOA4, AMBP, FABP1, and APOA2) were related to the survival differences of ECa.

**Conclusions:**

Our study reveals comprehensive clues that a novel signature based on CSRGs may provide reliable prognosis prediction and insight into new therapy for patients with ECa.

## Introduction

Esophageal carcinoma (ECa) is a highly aggressive malignancy and a healthcare problem with global impact. It ranks tenth in the incidence of malignancies worldwide and sixth in deaths from cancer (1). In 2020, 604,100 people (3.1% of new cancer cases) worldwide were diagnosed with ECa and 544,076 people (5.5% of new death cases) died from this disease (1). The course of treatment for ECa has changed significantly over the past decades. Early ECa is generally treated by endoscopy, and locally advanced ECa is routinely treated with neoadjuvant chemoradiotherapy before surgical treatment (2). However, for cervical ECa, chemotherapy and radiotherapy are the preferred treatment options (2). Despite the diversity of treatment options for ECa, the survival rates for ECa remains poor, mainly due to the late stage of the disease when first diagnosed and the high recurrence rate even in cases of localized disease. Consequently, it is vital for us to explore the appropriate therapeutic targets and novel prognostic biomarkers for ECa patients to enhance the clinical outcome.

Cellular senescence is characterized by a state of persistent cell cycle arrest in which cells remain merely metabolically active (3, 4). It is not only related to the aging process of organisms but also plays an important role in the whole life process from embryonic development to the end of life (5–7). One of the key features of senescent cells is the senescence-associated secretory phenotype (SASP), comprising three main features such as loss of proliferative or regenerative capacity, resistance to apoptosis, and accumulation of pathological metabolic wastes (8, 9). In recent years, more and more scholars have shown great interest in the intricate relationship between cellular senescence and cancers (10, 11). Previous studies have shown that cellular senescence acts as a double-edged sword at different stages of malignancy development (7, 12, 13). Consequently, abolishing accumulated deleterious cellular senescence and inducing acute cellular senescence are now being investigated as targets for treating disease. Recent research indicates that tumor cells might experience senescence as an evolutionary process, which involves both tumor intrinsic traits and external immunological load (14, 15). Notably, the negative consequences of SASP outweigh its positive features (16). Therefore, we hypothesized that with the accumulation of senescent cells, SASP remodels the tumor microenvironment by recruiting immunosuppressive cells, thereby promoting tumor cell evasion of immune surveillance, leading to poor clinical prognosis in tumors. To facilitate studies focused on cell senescence, the researchers developed CellAge, a database of genes associated with cell senescence. Developer manually-curated data is based on gene manipulation experiments in different human cell types. A gene expression signature of cellular senescence is also available. By integrating these and other datasets developers performed a systems biology analysis of cell senescence. At present, there are few studies on CSRGs in ECa, but a number of studies have shown that CSRGs signature can play a prognostic role in hepatocellular carcinoma, gastric cancer, bladder cancer, renal cell carcinoma, and colon cancer (17–21). However, the expression characteristics and prognostic significance of CSRGs in ECa remain unclear. Therefore, the study of cellular senescence in ECa is crucial.

In our study, a risk signature based on six CSRGs was constructed and validated in The Cancer Genome Atlas (TCGA) cohort and Gene Expression Omnibus (GEO) cohort, respectively. The actual prognostic value of risk signature in patients with ECa has also been fully explored. Based on the risk groupings, we next focused on the differences in clinical features, immune infiltration, and immunotherapy response between the two risk groups. Finally, we hope that our study can broaden the mind for prognostic prediction and individualizing immunotherapy of ECa.

## Materials and methods

### Data collection

**Figure 1:**
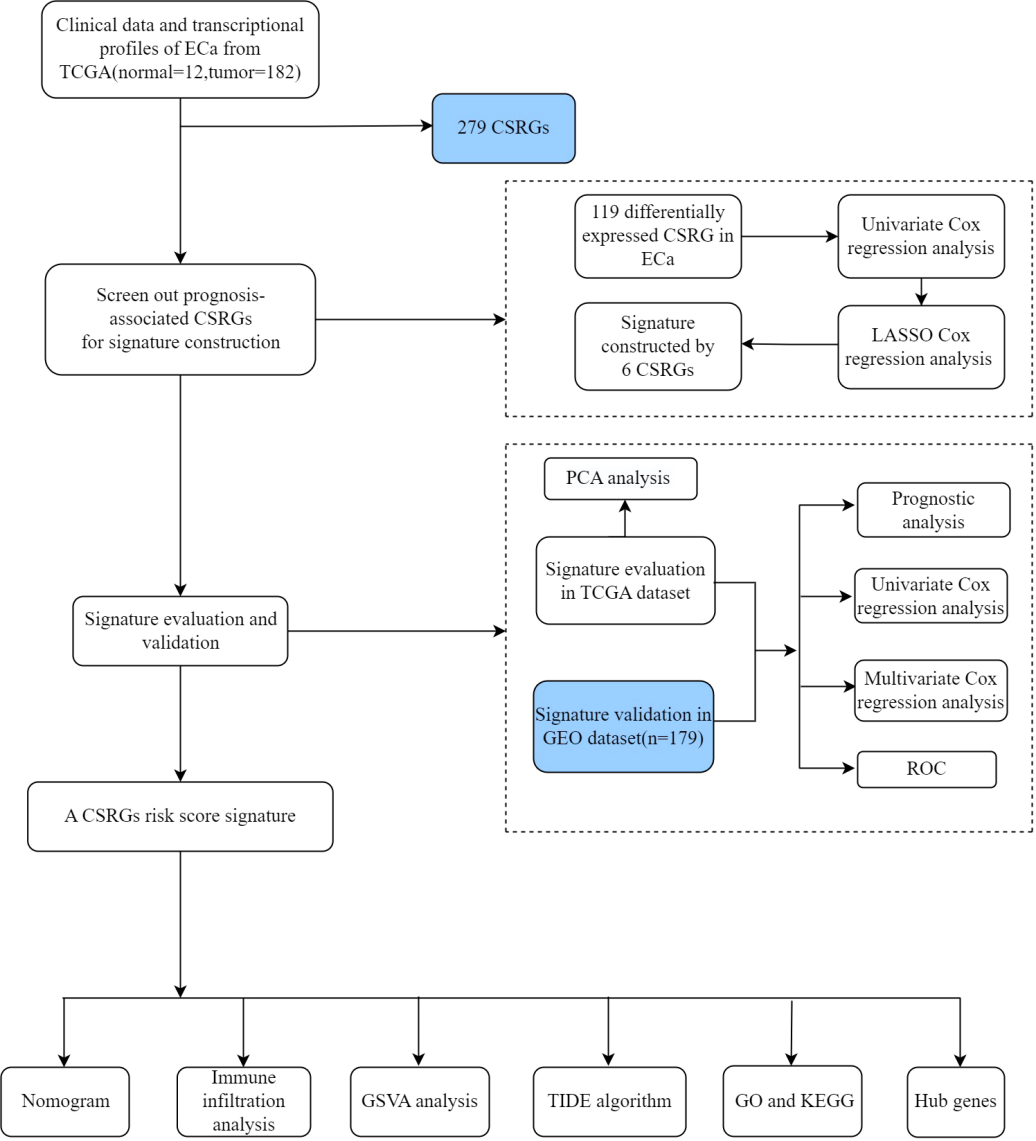
The detailed flow diagram in our study.

The gene expression profiles and clinical data of patients with ECa were extracted from the TCGA public database (https://portal.gdc.cancer.gov) and used as the training cohort. A total of 194 patients (12 normal esophageal tissue samples and 182 ECa samples) were included in the TCGA database. Independent probe matrix file (GSE53624) and platform file (GPL18109) containing 179 samples were derived from the GEO database (https://www.ncbi.nlm.nih.gov/geo/) and served as validation sets. According to the probe sequence of the platform file, the gene names were obtained by chip re-annotation technology, to obtain the corresponding relationship between the probe matrix and the gene names. Finally, we selected a list of 279 CSRGs (**Supplementary Table S1**) from the CellAge database (https://genomics.senescence.info/cells/). The flow diagram of this study is depicted in **Figure 1**.

### Identification of differentially expressed CSRGs in ECa

The expression differences of CSRGs between 12 normal esophageal tissues and 182 ECa tissues were analyzed by the “limma” R package. The data were analyzed strictly according to the screening criteria of false discovery rate (FDR)<0.05 and |log_2_ (fold change, FC)|>0.585. Next, differentially expressed CSRGs were visualized by plotting heat map and volcano map with the “pheatmap” package of the R software system.

### Development and verification of a prognostic-related CSRGs signature

We first combined the expression data in TCGA-ECa with the survival data and then analyzed and obtained the expression levels of differentially expressed CSRGs in ECa samples. Then, the CSRGs related to the prognosis of ECa were obtained by univariate Cox regression analysis, and the screening criteria was *P*<0.05. Finally, the Least Absolute Shrinkage and Selection Operator (lasso) regression analysis with the “glmnet” package in R was executed to pick up the preliminary hub CSRGs. The calculation formula of CSRGs-related risk scores was as follows: risk scores =Σ_i_(Lasso_Coef_i_*GeneExp_i_). “Lasso_Coef”, Lasso regression coefficient; “GeneExp”, amount of gene expression. According to the median risk score derived from CSRGs, patients with ECa were divided into high- and low-risk groups.

The effectiveness of the risk signature was demonstrated by principal component analysis (PCA), Kaplan–Meier survival curves, and receiver operating characteristic (ROC) curves. In order to verify the independence of the risk signature in prediction, univariate and multivariate Cox analyses on clinical variables and risk scores were generated using the “survival” package of R. The selected clinical variables are mainly indicators that have important prognostic significance for patients with ECa, such as age, gender, clinicopathological grade, and TNM stage. The “ggpubr” package of R was used to investigate whether there were differences in clinical variables and immune subtypes among different risk groups.

### RNA isolation and quantitative real-time PCR

Total RNA was extracted from 12 paired human ECa tissues and adjacent non-tumorous esophageal tissues using TRIzol Reagent (Abcam, China). The reverse transcription was conducted with TransScrip All-in-One SuperMix for qPCR reagent Kit (TaKaRa, Japan). Real-time fluorescent PCR was performed by SYBR Green assay. The experiment adopted a 20μL reaction system, Tip Green qPCR SuperMix (TaKaRa, Japan), cDNA template, upstream and downstream primers were successively added into 8 reaction tubes, and 3 repeated experiments were performed for each sample. We used glyceraldehyde-3-phosphate dehydrogenase (GAPDH) as the internal reference, and the data were analyzed using the 2^–ΔΔCt^ approach. The sequences of primers were listed in **Supplementary Table S2**.

### Construction of a nomogram

In order to predict the survival rate of patients with ECa at 1, 2, and 3 years, clinicopathological factors and risk score integrated nomogram was generated using the”regplot” and “rms” packages in R. Subsequently, by generating a calibration curve, we evaluated the preliminary consistency between the survival rate of ECa predicted by the nomogram and the actual survival rate. The ROC curve was utilized to explore the accuracy of the nomogram and clinical features in predicting the survival rate of patients with ECa. At last, univariate and multivariate Cox analyses were utilized to explore the potential of the nomogram to independently predict prognosis in ECa.

### Investigation of the immunotherapy response

R software combined with the CIBERSORT algorithm was used to explore the differences of 22 kinds of human immune cell subpopulations between the two risk groups in TCGA-ECa. Immediately after, we explored the differences in pathway enrichment between the two risk groups by gene set variation analysis (GSVA). The TIDE (Tumor Immune Dysfunction and Exclusion) algorithm was used to evaluate the weight of immunological rejection in two risk groups via an online website (http://tide.dfci.harvard.edu/).

### Functional and pathway enrichment analysis

Firstly, the “limma” package of R software was used to screen the differentially expressed genes (DEGs) between different groups, and the screening criteria were FDR<0.05 and |log_2_FC|>1. Subsequently, we performed GO and KEGG analysis on the two risk groups to explore the differences in their potential biological functions and pathways.

### Gene set enrichment analysis

Using the curated gene set (kegg.v7.4.symbols.gmt), broad GSEA v.4.2.3 was applied to detect high- and low-risk group correlation pathways with the criteria: NOM *P*<0.05 and |NES|>1 (22).

### Construction of the PPI network

The STRING online database (https://string-db.org/) was first applied to obtain the PPI (protein-protein interaction) information (interaction score >0.70) of the DEGs between different groups. Next, we visualized the PPI network using the Cytoscape software (version 3.9.1). In addition, the plugin of cytoHubba in Cytoscape was utilized to screen the hub genes with the most complex connections in the PPI network. Finally, the clinical significance of hub genes in ECa was further explored.

### Statistical analysis

Data processing, statistical analysis, and diagram formation were all conducted in R software (version R-4.2.2), GraphPad Prism (version 9.0), and Cytoscape software (version 3.9.1). The Kaplan–Meier curve plotted by the “survminer” package of R was used to compare differential survival probability. Univariate and multivariate Cox regression analyses of independent prognostic factors were performed using the “forestplot” package of R. ROC curve plotted by the “timeROC” package of R was used to assess the predictive efficacy of the CSRGs prognostic signature and nomogram. Results with two-sided *P*<0.05 were deemed statistically significant.

## Results

### Identification of differential CSRGs in ECa

**Figure 2:**
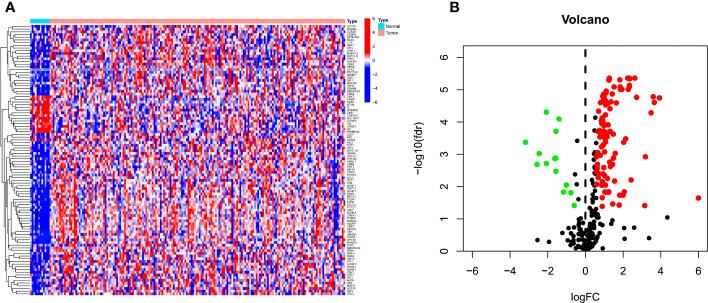
**(A, B)** Heat map and volcano map of differential CSRGs in ECa and normal esophageal tissues. Green, downregulated; red, upregulated.

In TCGA data, among 279 CSRGs, 119 were differentially expressed between ECa samples and normal esophageal samples (FDR <0.05, |log_2_FC| > 0.585), including 15 downregulated genes and 104 upregulated genes in the ECa tissue samples. These above differences are displayed in the heat map and volcano map (**Figures 2A, B**).

### Construction of a prognostic signature and signature gene analysis

**Figure 3:**
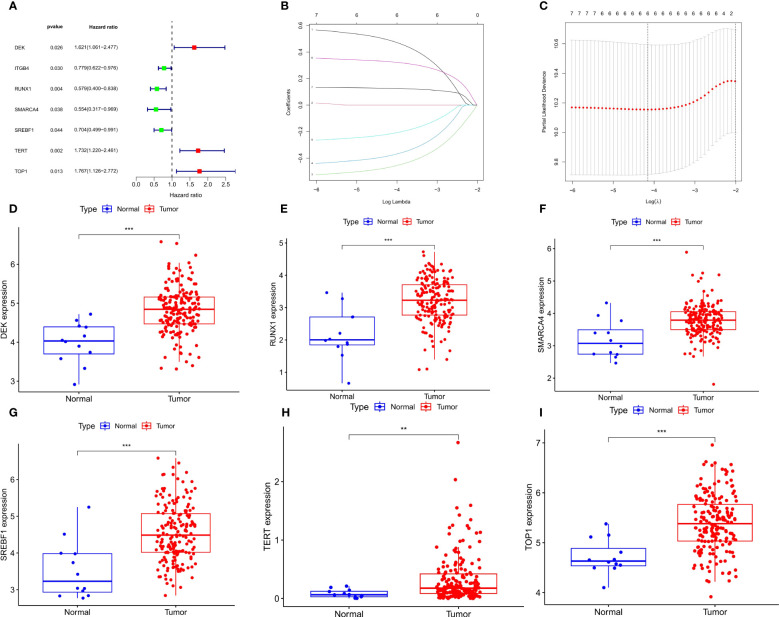
Develop a prognostic signature and signature gene analysis. **(A)** Forest plot. The 7 CSRGs associated with ECa prognosis. **(B)** LASSO coefficient profiles of 7 CSRGs. **(C)** The numbers on the graph represent the number of genes associated with the prognosis of ECa; Cross-validation for tuning parameter selection to filter the key genes. **(D-I)** Expressed divergence of signature genes between ECa tissues and normal esophageal tissues. **P < 0.01, *** P < 0.001.

Based on univariate Cox regression analysis of the TCGA-ECa cohort, 7 CSRGs that significantly affect the prognosis of ECa were disclosed, such as TERT (**Figure 3A**). Subsequently, in order to develop a CSRGs signature for survival prediction of ECa, the 6 OS (overall survival)-associated CSRGs were analyzed using the LASSO analysis. Finally, a total of 6 genes (DEK, RUNX1, SMARCA4, SREBF1, TERT, TOP1) were built (**Figures 3B, C**). The risk scores for all patients were calculated as follows: risk score = (0.486102936551265×DEK level) + (-0.466577382724787×RUNX1 level) + (-0.379803436002195×SMARCA4 level) + (-0.216260668499574×SREBF1 level) + (0.326358456240447×TERT level) + (0.126266167208409×TOP1 level) (**Supplementary Table S3**). The results of our further analysis of the expression levels of signature genes in ECa and normal esophageal tissues are as follows. Compared with the normal esophageal tissues group, DEK, RUNX1, SMARCA4, SREBF1, TERT, and TOP1 all showed elevated expression abundance in the ECa tissues group (**Figures 3D-I**).

### Validating the expression levels of signature genes in ECa patients

**Figure 4:**
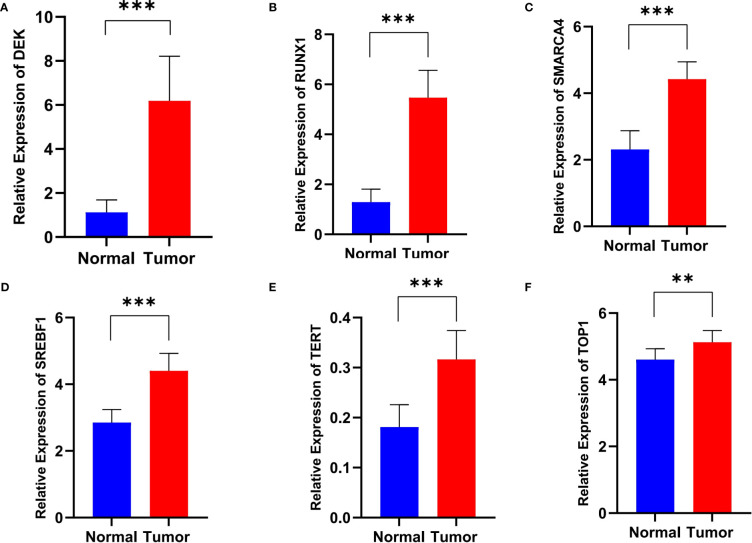
The relative RNA level of DEK **(A)**, RUNX1 **(B)**, SMARCA4 **(C)**, SREBF1 **(D)**, TERT **(E)**, and TOP1 **(F)** in ECa tissues and adjacent normal esophageal tissues. Data are presented as Mean with SD, **P < 0.01, *** *P* < 0.001.

Real-time fluorescence quantitative PCR (RT-qPCR) was performed to verify the mRNA level of the six signature genes in 12 paired ECa tissues and adjacent normal esophageal tissues. The results indicated that the expression of DEK, RUNX1, SMARCA4, SREBF1, TERT, and TOP1 were all up-regulated in ECa tissues than that in adjacent normal tissues (**Figures 4A-F**). These results were consistent with the expression tendencies of the previous signature genes in esophageal cancer and normal esophageal tissues.

### Validation of a CSRGs prognostic signature

**Figure 5:**
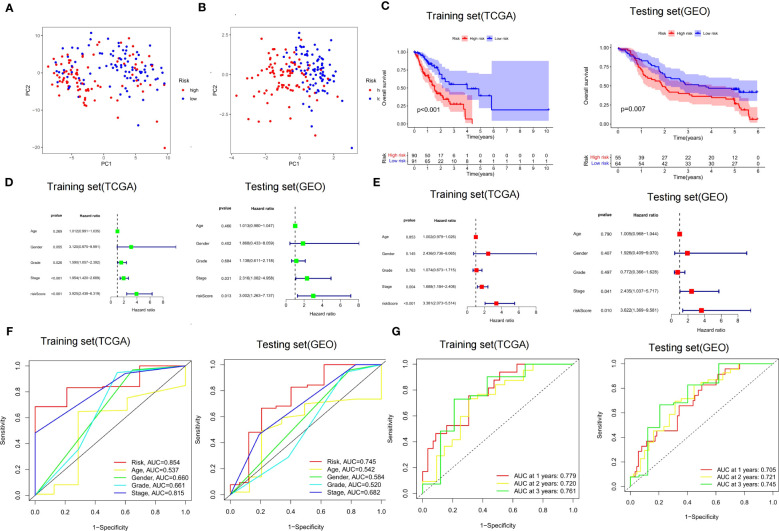
Validation of risk score signature and implications for prognosis. **(A, B)** PCA chart. High- and low-risk groups were differentiated by CSRGs and signature genes, respectively. **(C)** K-M curves for the OS of the prognostic signature in the TCGA-ECa cohort and GEO-ECa cohort, respectively. **(D, E)** Univariate and multivariate Cox regression analyses of prognostic factors in TCGA-ECa cohort and GEO-ECa cohort, respectively. **(F)** The AUC values of the risk signature were the highest in the TCGA-ECa cohort and GEO-ECa cohort. **(G)** ROC analysis was employed to evaluate the capacity of the risk signature in OS in the TCGA-ECa cohort and GEO-ECa cohort.

It was found by PCA that our constructed CSRGs signature can accurately divide ECa samples into high- and low-risk groups (**Figures 5A, B**). Similar to the results obtained from the training cohort, patients in the high-risk group of the testing cohort were more likely to encounter a worse prognosis (**Figure 5C**). The results of univariate Cox regression analysis combined with multivariate Cox regression analysis in the TCGA-ECa cohort and GEO-ECa cohort suggested that the risk score based on CSRGs remained an independent risk factor affecting the prognosis of ECa (**Figures 5D, E**). In predicting survival, the risk score had a larger area under the ROC curve than other clinical features in the TCGA-ECa cohort and GEO-ECa cohort, suggesting that the risk score could serve as a more accurate prognostic factor (**Figure 5F**). Afterward, ROC analysis was employed to assess the risk signature in OS, with AUC values of 0.779, 0.720, and 0.761 at the 1-, 2-, and 3-year in TCGA-ECa cohort and 0.705, 0.721, and 0.745 at the 1-, 2-, and 3-year in the GEO-ECa cohort, respectively (**Figure 5G**).

### Clinicopathological characteristics and prognostic value in different risk groups

**Figure 6:**
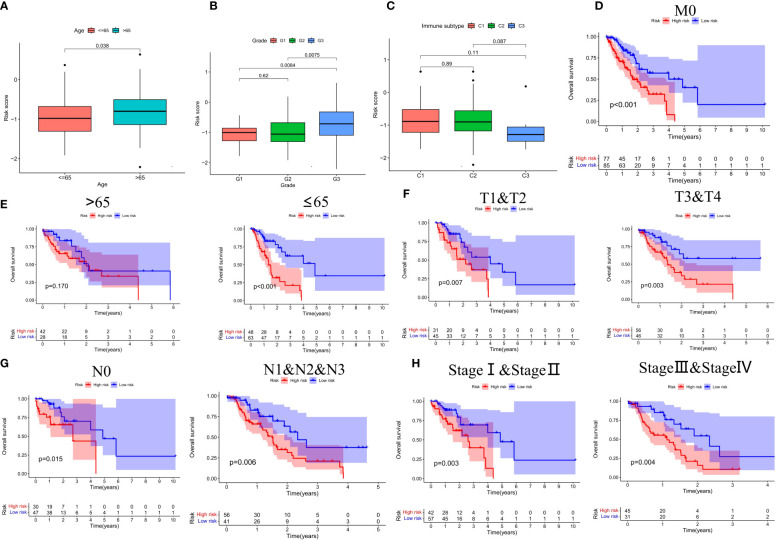
**(A-C)** Box plots of different risk groups at different ages, grades and, immune subtypes. Subgroup survival analysis of M0 stage **(D)**, age **(E)**, T stage **(F)**, N stage **(G)**, and pathological staging **(H)** between high- and low-risk score groups.

Based on the results of risk grouping, we are interested in whether there are differences in clinical features among different risk groups and conducting further analysis. We found a significantly increased risk in patients older than 65 years and in patients with grade G3 (**Figures 6A, B**). However, other clinical features did not show statistical significance between the different risk score groups (**Supplementary Figure S1**). Thorsson et al. performed an extensive immunogenomic analysis of over 10,000 tumors comprising 33 diverse cancer types utilizing data compiled by TCGA (23). Across cancer types, they identified six immune subtypes by RNA sequencing: C1-Wound Healing, C2-IFN-γ Dominant, C3-Inflammatory, C4-Lymphocyte Depleted, C5-Immunologically Quiet, and C6-TGF-β Dominant (23). Next, we also found that the risk score between the different immune subtypes was also not statistically different (**Figure 6C**). Finally, we investigated the prognostic value of CSRGs risk score signature in different subgroups of patients with ECa (**Figures 6D–H**). CSRGs risk score can accurately determine prognosis in ECa patients with either aged less than 65 years (*P*<0.001) or M0 stage (*P*<0.001), as well as in patients with ECa in T1&T2 stages (*P*=0.007) or T3&T4 stages (*P*=0.003) or N0 stage (*P*=0.015) or N1&N2&N3 stages (*P*=0.006) or pathological stage I & II (*P*=0.003) or pathological stage III & IV (*P*=0.004). However, CSRGs risk score signature was not a good predictor of prognostic outcome for ECa patients aged more than 65 years (*P*=0.170).

### Construction of a clinical nomogram

**Figure 7:**
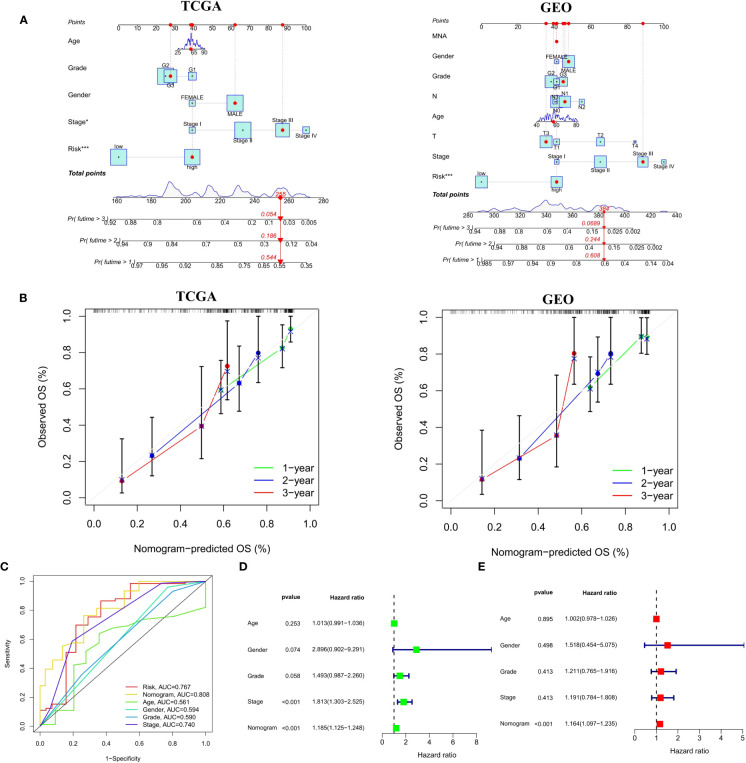
Construction and verification of a Nomogram. **(A)** Nomogram for forecasting the 1-, 2-, and 3-year mortality in the TCGA-ECa cohort and GEO-ECa cohort. **(B)** Calibration curve of the prediction efficiency of Nomogram in TCGA-ECa cohort and GEO-ECa cohort. **(C)** The AUC value of the nomogram was the highest at 0.808 in the TCGA cohort. **(D, E)** Univariate and multivariate Cox regression analyses of prognostic factors associated with OS in TCGA cohort, including Nomogram.

In order to broaden the application of the CSRGs in ECa patients, we developed a quantitative nomogram to compute OS at 1, 2, and 3 years in the TCGA-ECa cohort and GEO-ECa cohort (**Figure 7A**). In the TCGA-ECa cohort, when the prognostic parameters’ point is 255, the predicted OS of patients with ECa is 0.544 at 1 year, 0.186 at 2 years, and 0.054 at 3 years. The OS prediction lines of the nomogram are close to the 45° standard curve of the calibration analysis in the TCGA-ECa cohort and GEO-ECa cohort, demonstrating that the established clinical nomogram performs excellent (**Figure 7B**). It confirmed the high predictive efficiency of the nomogram in OS of patients with ECa. We also found that the AUC value of the nomogram in the ROC curve reached 0.808, suggesting that the nomogram was a better predictor of survival than other prognostic indicators of ECa patients in the TCGA cohort (**Figure 7C**). In addition, the results of univariate Cox regression analysis combined with multivariate Cox regression analysis (**Figures 7D, E**) suggested that the nomogram (HR=1.164, 1.097-1.235, *P*<0.001) based on risk score remained an independent risk factor affecting the prognosis of ECa in TCGA cohort.

### Relationship of the CSRGs signature with tumor immunotherapy

**Figure 8:**
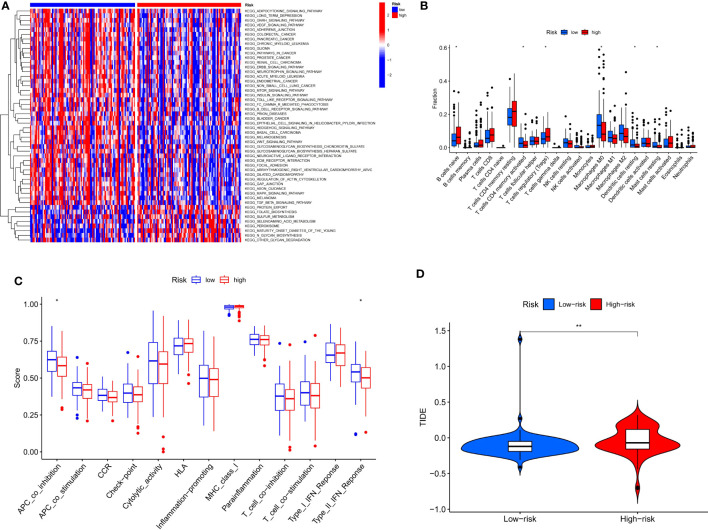
Risk score guides immunotherapy. **(A)** Heat map of GSVA analysis for two risk groups. **(B, C)** Box plot of immune signature analysis between two risk groups. **(D)** Violin plot of TIDE score distribution between two risk groups. * P < 0.05, **P < 0.01.

By performing GSVA (Gene Set Variation Analysis), we evaluate the relative expression difference of the pathways between two risk groups. Many differentially expressed pathways were enriched by GSVA analysis and finally visualized by heatmap (**Figure 8A**). Compared with the low-risk group, the expression of pathways associated with metabolism and transport of cellular processes were remarkably activated in the high-risk group, whereas the expression of tumor and genetic information processing associated pathways were significantly lower. Analysis of immune cell infiltration revealed higher naïve B cells and regulatory T cells (Tregs) in the high-risk group, however, activation of M0 macrophages and activated memory CD4+ T cells was higher in the low-risk group (**Figure 8B**). In addition, compared with the high-risk group, immune function analysis displayed that APC_co_inhibition and type_II_IFN_response were more activated in the low-risk group (**Figure 8C**). Finally, the TIDE algorithm showed that the high-risk group has a greater risk of immune escape during immunotherapy, which also implied that the low-risk group of ECa patients may benefit from immunotherapy (**Figure 8D**).

### Functional enrichment analysis and gene set enrichment analysis

**Figure 9:**
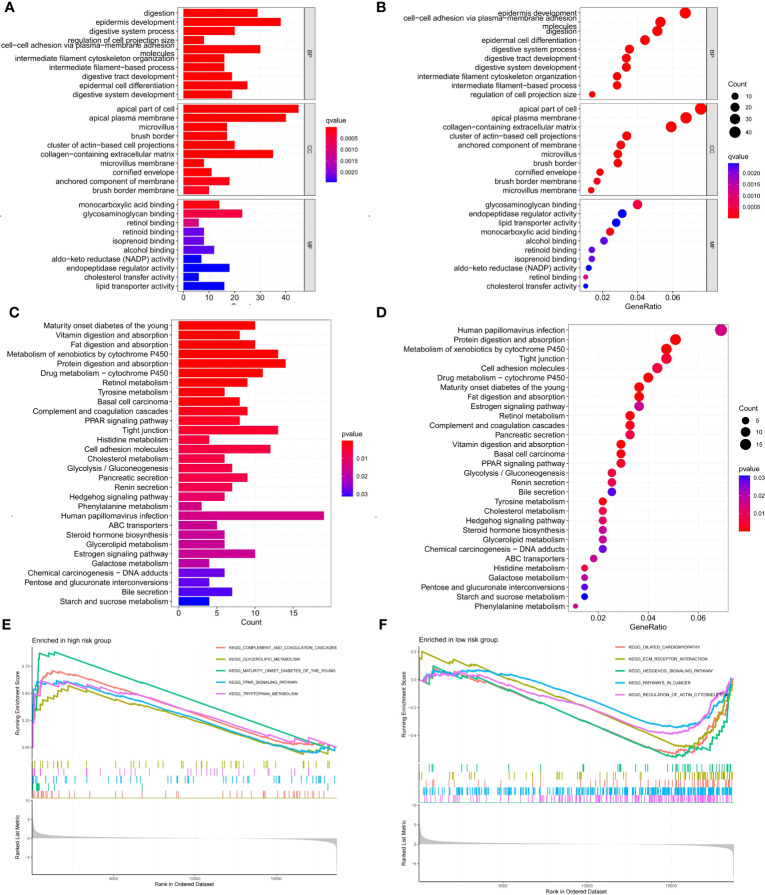
Enrichment analysis of DEGs between two risk groups and GSEA analysis of 6 signature genes in high- and low-risk groups. **(A, B)** GO enrichment analysis of DEGs between two risk groups. **(C, D)** KEGG enrichment analysis of DEGs between two risk groups. **(E, F)** GSEA analysis of the top five pathways enriched by 6 signature genes in high- and low-risk groups.

We screened 747 DEGs between the two risk groups. GO and KEGG analysis further elucidated differences in biological functions and pathways between the two risk groups. The biological process (BP) modules of GO analysis are mainly focused on epidermis development, digestion, epidermal cell differentiation, digestive system process, and so on (**Figures 9A, B**). However, the human papillomavirus infection, protein digestion and absorption, metabolism of xenobiotics by cytochrome P450, and tight junction pathways were observably enriched in the KEGG analysis (**Figures 9C, D**). To explore the different biological functions of 6 cellular senescence-related signature genes in two risk groups, the GSEA (gene set enrichment analysis) analysis was used to identify the top five pathways. GSEA analysis showed that complement and coagulation cascades, glycerolipid metabolism, maturity-onset diabetes of the young, PPAR signaling pathway, and tryptophan metabolism were enriched in high-risk groups (**Figure 9E**). However, dilated cardiomyopathy, ECM receptor interaction, hedgehog signaling pathway, pathways in cancer, and regulation of actin cytoskeleton were enriched in low-risk groups (**Figure 9F**).

### Identification of 10 hub candidate genes with the PPI network

**Figure 10:**
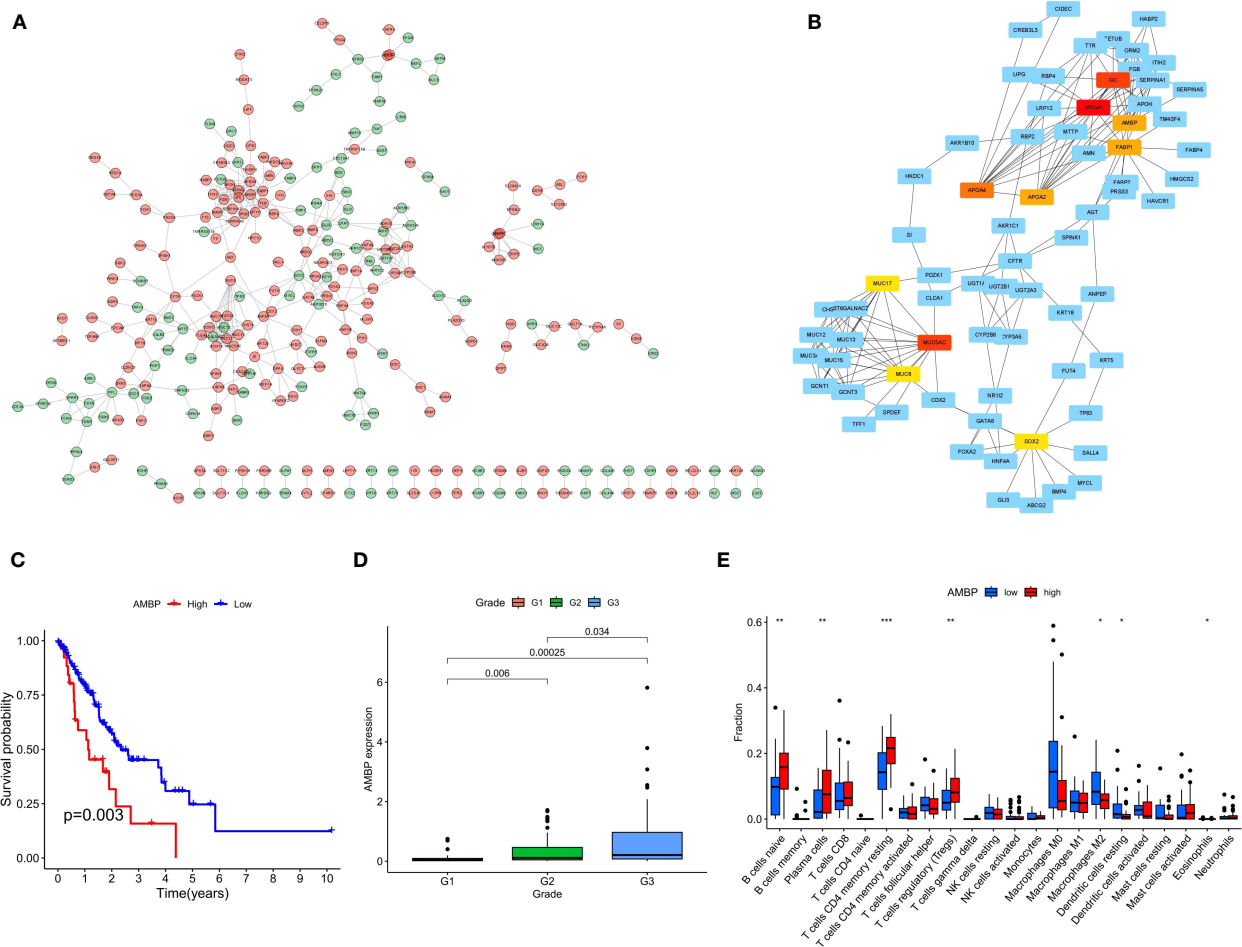
The PPI network complex for the DEGs and expression levels of hub genes among different clinical characteristics. **(A)** Proteins encoded by 293 DEGs were used to construct the PPI network. **(B)** A total of 10 hub genes were identified using Cytoscape. **(C)** K-M curves based on AMBP level in ECa patients. **(D)** Box plot of the relationship between the expression of AMBP and different grades. **(E)** Box plot of the relationship between the expression of AMBP and different immune cells. * P < 0.05, ** P < 0.01, *** P < 0.001.

In order to screen the differential hub genes that are involved in two risk score groups from the interaction level, the expression profiles of DEGs were visualized by the STRING database and Cytoscape software. Proteins encoded by 293 differential genes were used to construct the PPI network, which included the interaction relationship of 177 up-regulated genes and 116 down-regulated genes (**Figure 10A**). Finally, the 10 hub genes (APOA1, MUC5AC, GC, APOA4, AMBP, FABP1, APOA2, SOX2, MUC8, MUC17) with the highest interaction degrees were identified by Cytoscape (**Figure 10B**). By further analysis, 4 hub genes (APOA4, AMBP, FABP1, and APOA2) with survival differences were identified (**Figure 10C**; **Supplementary Figure S2**). The results demonstrated that patients with ECa with high expression of the above 4 genes had a lower probability of survival.

We further analyzed the relationship between the expression level of these 4 genes and the clinical features of ECa. In ECa patients with grade G2 and G3, the expression level of APOA4 was significantly higher than that in grade G1 patients (**Supplementary Figure S3**). With the increase of G grade level in patients with ECa, the expression of AMBP gradually increased with statistical significance (**Figure 10D**). In ECa patients over 65 years old, the FABP1 expression level was significantly higher than that below 65 years old (**Supplementary Figure S4**). Finally, the relationship between the expression levels of these 4 genes and different immune cells was further explored. The AMBP and FABP1 high expression groups had higher resting CD4 memory T cells infiltration, suggesting that those groups of patients are more suitable for immunotherapy (**Figure 10E**; **Supplementary Figure S5**).

## Discussion

Because patients with ECa do not feel any discomfort early on, the prognosis of patients with ECa is relatively poor. In the past diagnosis of ECa, early-stage cancer only accounts for about 5% (24). Due to the strong aggressiveness of ECa, when symptoms such as dysphagia appear, most of the patients are already at an advanced stage, resulting in a very poor prognosis for patients. However, despite the diversity of treatment options for ECa, the 3- and 5-year survival rates of patients fluctuate between 6% to 35% (24). Although the treatment strategy and individualized treatment of patients with ECa have improved, a considerable proportion of patients who receive comprehensive treatment still gained disappointing improvements in survival (25). It can be seen that the current effective clinical treatment for patients with ECa is quite limited. Therefore, it is greatly important to develop models for optimizing decision-making strategies for ECa management.

Cellular senescence refers to a state of persistent growth arrest induced by a variety of endogenous and exogenous stress (26). Accumulative evidence points to several common hallmarks of cellular senescence including high expression of the cell cycle inhibitor p16Ink4a, and a unique SASP involving matrix metalloproteinases, cytokines, growth factors, chemokines, and angiogenic factors (27). An increasing number of investigations have indicated that cellular senescence plays a considerable role in tumor microenvironment remodeling and tumor proliferation (28, 29). Considering that there are few studies on cellular senescence in ECa, this study explores the value of CSRGs in the prognosis and treatment of ECa. Finally, six genes, including DEK, RUNX1, SMARCA4, SREBF1, TERT, and TOP1 were filtered out to construct the prognostic signature. Intriguingly, previous studies have found that these landmark genes have been identified as playing an important role in the biological processes of various malignancies.

DEK encodes a protein consisting of 275 amino acids with a molecular weight of about 43 kDa (30). Multiple studies have shown that DEK is upregulated in a variety of malignant conditions, such as acute myelocytic leukemia(AML) (31–33), melanoma (34), hepatocellular carcinoma (35), retinoblastoma (36, 37), urinary bladder cancer (38, 39), glioblastoma (40), and oral squamous cell carcinoma (SCC) (41). Matrka et al. revealed for the first time that overexpression of DEK in mice contributes to an increase in the overall incidence of ECa and a trend toward increased cell proliferation was detected in adjacent normal esophageal tissues (42). There are three RUNX (including RUNX1, 2, and 3) family members in mammals, and different RUNX proteins have different tissue-specific expressions and exhibit different biological significance (43). Many studies in the past have confirmed that RUNX1 played a central role in epithelial tumorigenesis through the RUNX1-Stat3 axis (44, 45). SMARCA4 is considered to play a critical role in cell growth arrest and cellular senescence and is hypothesized to be a tumor suppressor gene in lung cancer (46, 47). SREBF1 has been shown to have a strong tumorigenic role in many malignant types including hepatocellular carcinoma, prostate cancer, and breast cancer (48). Li et al. also found that SREBF1 is an underlying therapeutic target and prognostic indicator in ECa (49). TERT gene, located in human chromosome 5p15.33, is the catalytic subunit of telomerase, is an indispensable and important part of telomerase-holoenzyme, and can play a crucial role in the formation of carcinoma through telomere-dependent or independent mechanism (50). Topoisomerase 1 (TOP1) can release topological stress due to natural processes such as replication and transcription and is an essential enzyme for life processes (51). If endogenous or exogenous DNA damage is not repaired by TOP1, it will ultimately contribute to cell death resulting from the accumulation of cytotoxic double-strand breaks (DSB) (52, 53). This regulatory mechanism has also been used in the anti-cancer treatment of various tumors, such as colorectal cancer (54), lung cancer (55), and ovarian cancer (56). Based on the evidence of the above findings, it further indicates that CSRGs may predict the prognosis of ECa.

By searching the TCGA database, we developed a prognostic risk signature for patients with ECa using CSRGs. In order to comprehend the underlying function of the signature in ECa, PCA analysis was first performed by us. The results revealed that patients with ECa could be more accurately segmented into two risk groups based on six CSRGs, which further proved the superiority of the signature. Survival analysis demonstrated that patients in the high-risk score group had a significantly poorer OS. We further rebuilt the signature with the same criteria in the validation cohort to verify the stability of the previously constructed risk signature. As expected, consistent with the prognostic result obtained in the TCGA, patients with higher risk scores of the GEO also exhibited worse OS. Furthermore, the results of multivariate Cox analysis verified that the risk signature based on CSRGs remained an independent risk factor affecting the prognosis of ECa in the TCGA-ECa cohort and GEO-ECa cohort. The CSRGs risk score can accurately determine prognosis in ECa patients with either aged less than 65 years or M0 stage, as well as in patients with ECa in T1&T2 stages or T3&T4 stages or N0 stage or N1&N2&N3 stages or pathological stage I & II or pathological stage III & IV. These results further demonstrate the practicability of our signature in the clinic. Immediately afterward, in order to broaden the application of the CSRGs in ECa patients, we developed a quantitative nomogram to compute OS at 1, 2, and 3 years. The OS prediction lines of the nomogram are close to the 45° standard curve of the calibration analysis, demonstrating that the established clinical nomogram performs excellent. Encouragingly, we also found that the AUC value of the nomogram in the ROC curve reached 0.808 in the TCGA cohort, suggesting that the nomogram was a better predictor of survival than other prognostic indicators of ECa patients. At last, the results of univariate Cox regression analysis combined with multivariate Cox regression analysis suggested that the nomogram based on risk score remained an independent risk factor affecting the prognosis of ECa.

With the development of medicine, more and more treatments are available for ECa (57). The core of tumor immunotherapy is to regulate the disordered immune function of the body, relying on the immune system to function to kill cancer cells and tumor tissues (58). This often leads to the abuse of immunotherapy drugs as it is currently unclear which patients with ECa benefit from immunotherapy in practice. Therefore, we conducted a further analysis using the available signature to distinguish which patients with ECa would benefit more from immunotherapy. Analysis of immune cell infiltration revealed higher regulatory T cells (Tregs) (tumor-promoting cells) in the high-risk group, however (59), activation of M0 macrophages and memory CD4+ T cells (anti-tumor cells) activated was higher in the low-risk group (60). In addition, compared with the high-risk group, immune function analysis displayed that APC_co_inhibition and type_II_IFN_response were more activated in the low-risk group. APC-co-inhibition describes an important mechanism of interaction between antigen-presenting cells (APC) and T cells. In this interaction, co-stimulatory molecules on the surface of antigen-presenting cells, such as CD80/86, bind to CD28 on the surface of T cells, initiating a T-cell immune response (61). Other molecules, such as CTLA-4, competitively bind CD80/86 to inhibit the T-cell immune response, known as APC-co-inhibition (61). Therefore, APC-CO-inhibition is not an independent immune pathway, but an immunomodulatory mechanism that can play a role in a variety of immune pathways, such as the TCR signaling pathway and NF-kB signaling pathway (61). The research results of Liu et al. revealed that “type_II_IFN_response” is an immune-related function associated with anti-tumor (62), which coincides with the results of our study. Based on these findings, we speculated that the low-risk group had a more effective response to immunotherapy than the high-risk group. Finally, the TIDE algorithm showed that the high-risk group had a greater risk of immune escape during immunotherapy, which also implied that the low-risk group of ECa patients may benefit from immunotherapy. On the whole, the prognostic signature of CSRGs that we constructed can not only predict the prognosis of ECa patients but also identify immunotherapy-sensitive patients.

In view of the significant differences in prognosis between the two risk groups, it is necessary to conduct in-depth research on the differential genes. We screened out 10 hub genes (APOA1, MUC5AC, GC, APOA4, AMBP, FABP1, APOA2, SOX2, MUC8, MUC17) by constructing a PPI network. Subsequently, by survival analysis, we observed that AMBP, APOA2, APOA4, and FABP1 were negatively correlated with the prognosis of ECa. At last, we also found higher immune infiltration (resting CD4 memory T cells) in the high-expression group of AMBP and FABP1, while macrophages M2 showed higher infiltration in the low-expression group of AMBP and FABP1. M2 macrophages, contrary to M1 cells that are pro-inflammatory and cytotoxic, are immunosuppressive and favor angiogenesis and tissue repair (63). Many studies have shown that tumor-associated M2 macrophages improve tumor cell growth and survival and stimulate angiogenesis and metastases (63, 64). A recent study has demonstrated that resting CD4 memory T cells were the protective factor for CRC (colorectal cancer) and could act as an independent prognostic factor based on a large sample analysis of 879 CRC patients (65). Moreover, some studies showed that resting CD4 memory T cells were associated with increased overall survival in various cancers (66, 67). It suggested that patients with high expression of AMBP and FABP1 might be more suitable for immunotherapy.

## Conclusion

In the present study, our work identified and validated a CSRGs signature with independent prognostic significance for patients with ECa. The prognostic signature based on CSRGs established in this study is helpful to predict the survival rate of patients with ECa and guide clinical treatment. Patients with a low-risk group of the CSRGs signature may have a better immunotherapy effect. Therefore, our findings might facilitate the understanding of cellular senescence in ECa and provide certain guiding significance for immunotherapy. However, the current signature should be further explored and may provide some new insights into the mechanisms behind CSRGs in ECa.

## Data availability statement

The datasets presented in this study can be found in online repositories. The names of the repository/repositories and accession number(s) can be found in the article/**Supplementary Material**.

## Ethics statement

The studies involving human participants were reviewed and approved by The First Affiliated Hospital of Anhui Medical University. The patients/participants provided their written informed consent to participate in this study. Written informed consent was obtained from the individual(s) for the publication of any potentially identifiable images or data included in this article.

## Author contributions

Experimental design: YW, and WW. Data collection: YW, LD, and RH. Methodology: YW, LD, WL, and WW. Writing-original draft: YW, LD, and WW. Writing-review and editing: YW, LD, RH, and WW. All authors read and approved the final manuscript. All authors contributed to the article and approved the submitted version.
