# Supplementary figures and images for: Prognosis signature for predicting the survival and immunotherapy response in esophageal carcinoma based on cellular senescence-related genes
Source: Front Oncol. 2023 Aug 17;13:1203351. doi: 10.3389/fonc.2023.1203351 (PMC10470646; doi:10.3389/fonc.2023.1203351)

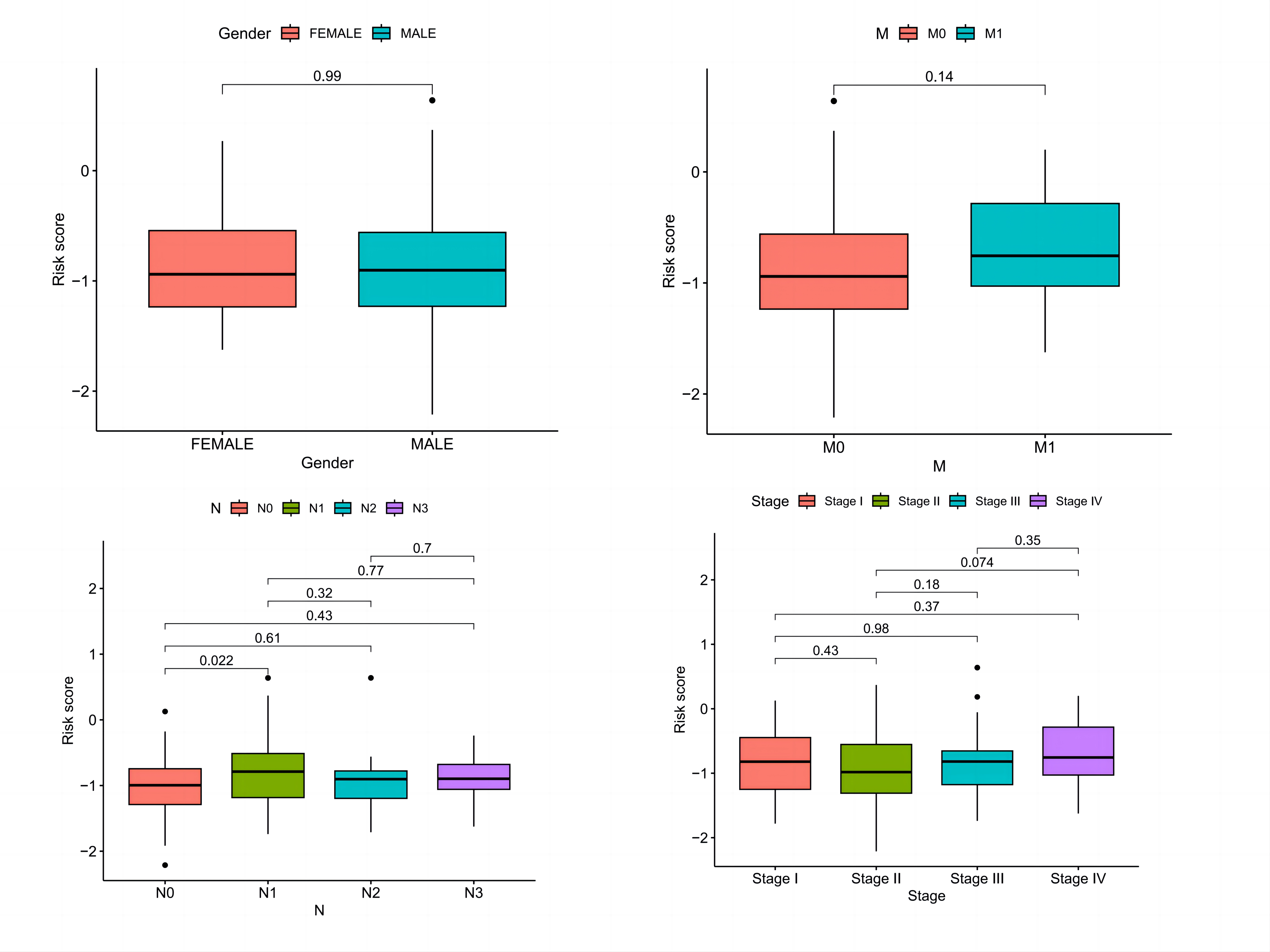

Supplement: Supplementary file 1 [file Image_1.jpeg]

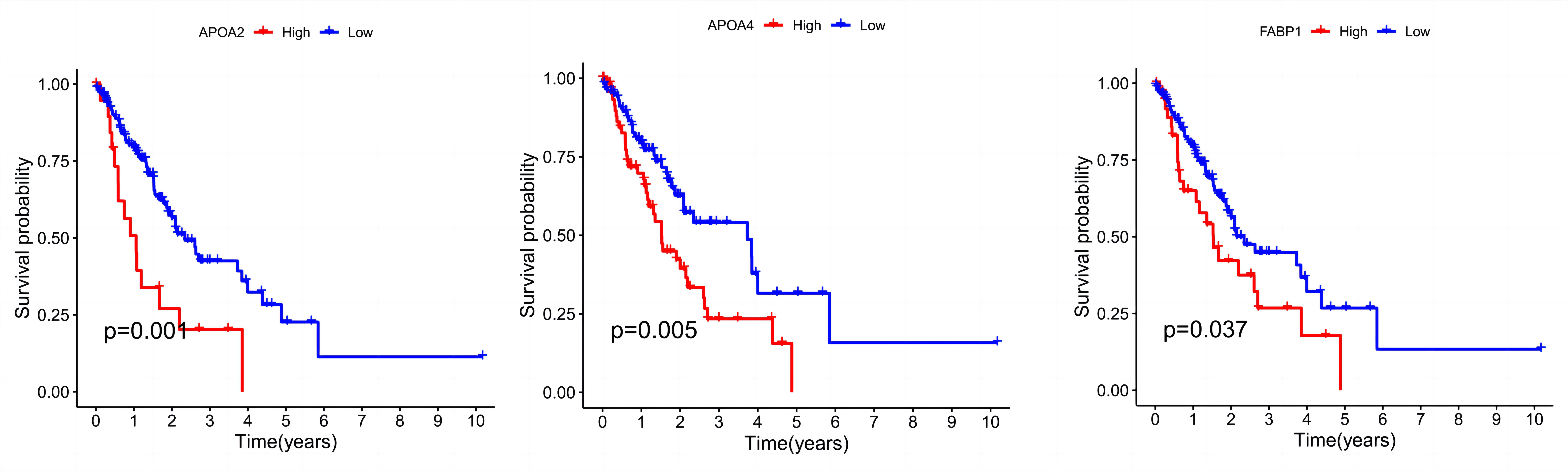

Supplement: Supplementary file 2 [file Image_2.jpeg]

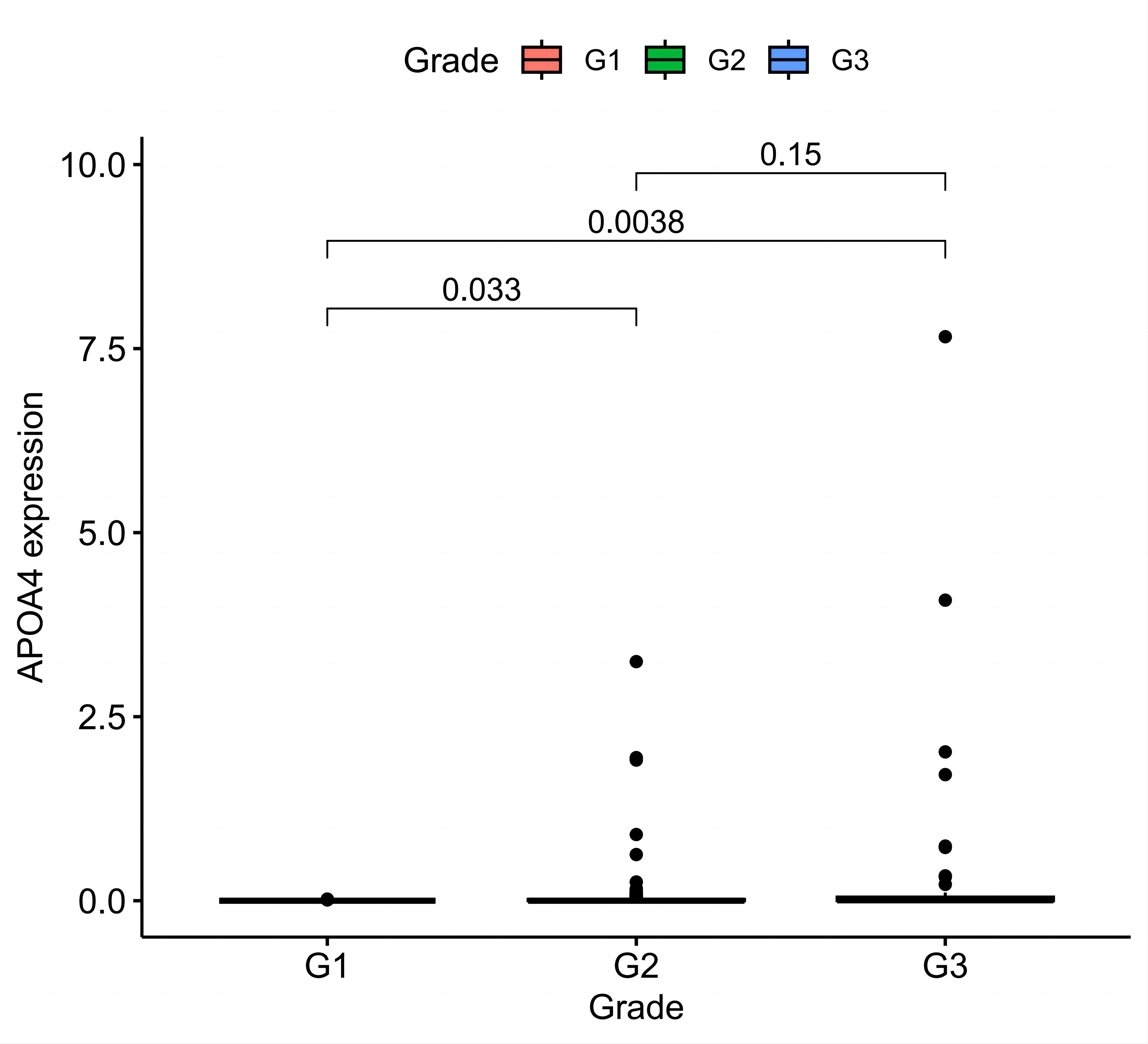

Supplement: Supplementary file 3 [file Image_3.jpeg]

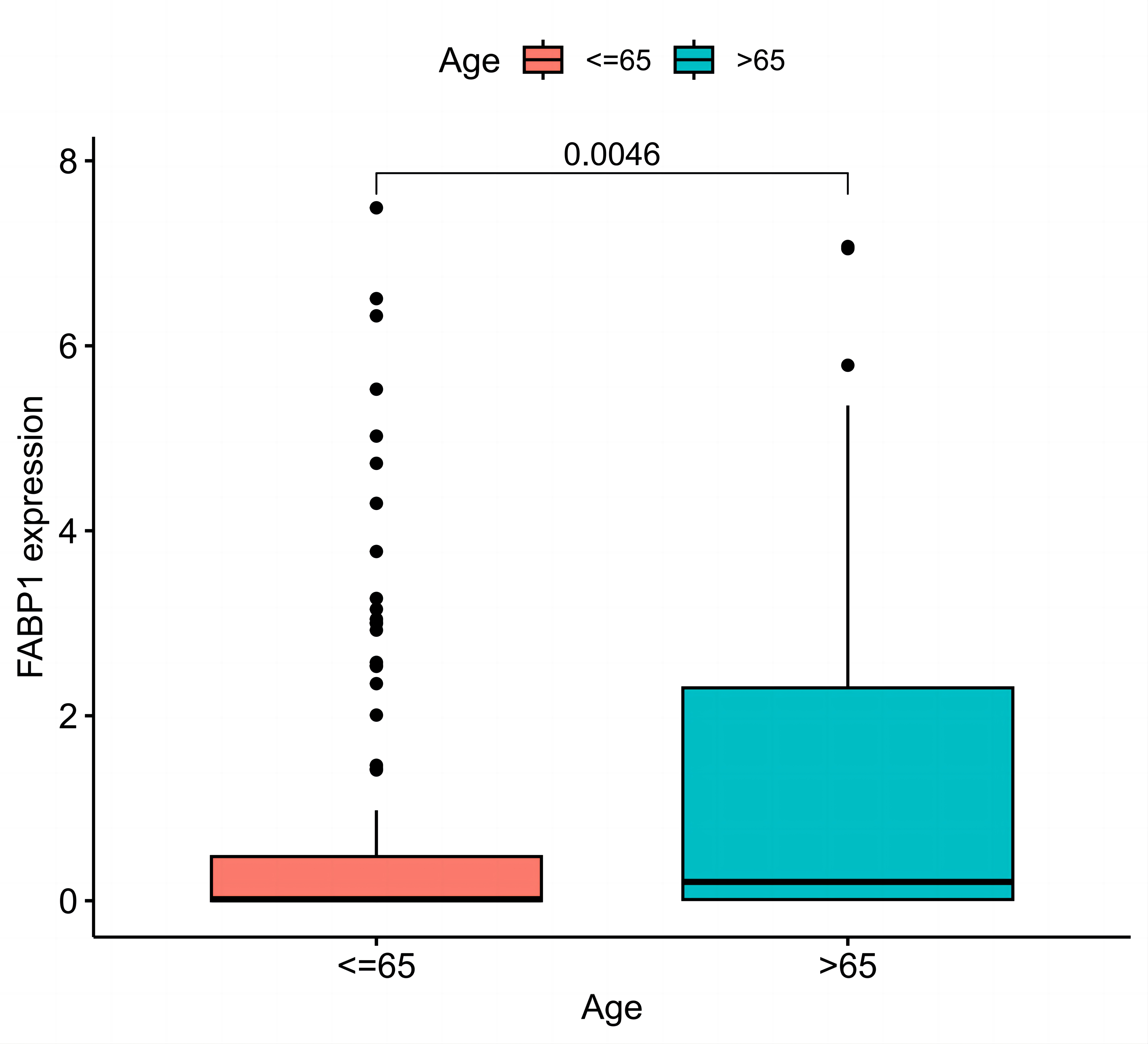

Supplement: Supplementary file 4 [file Image_4.jpeg]

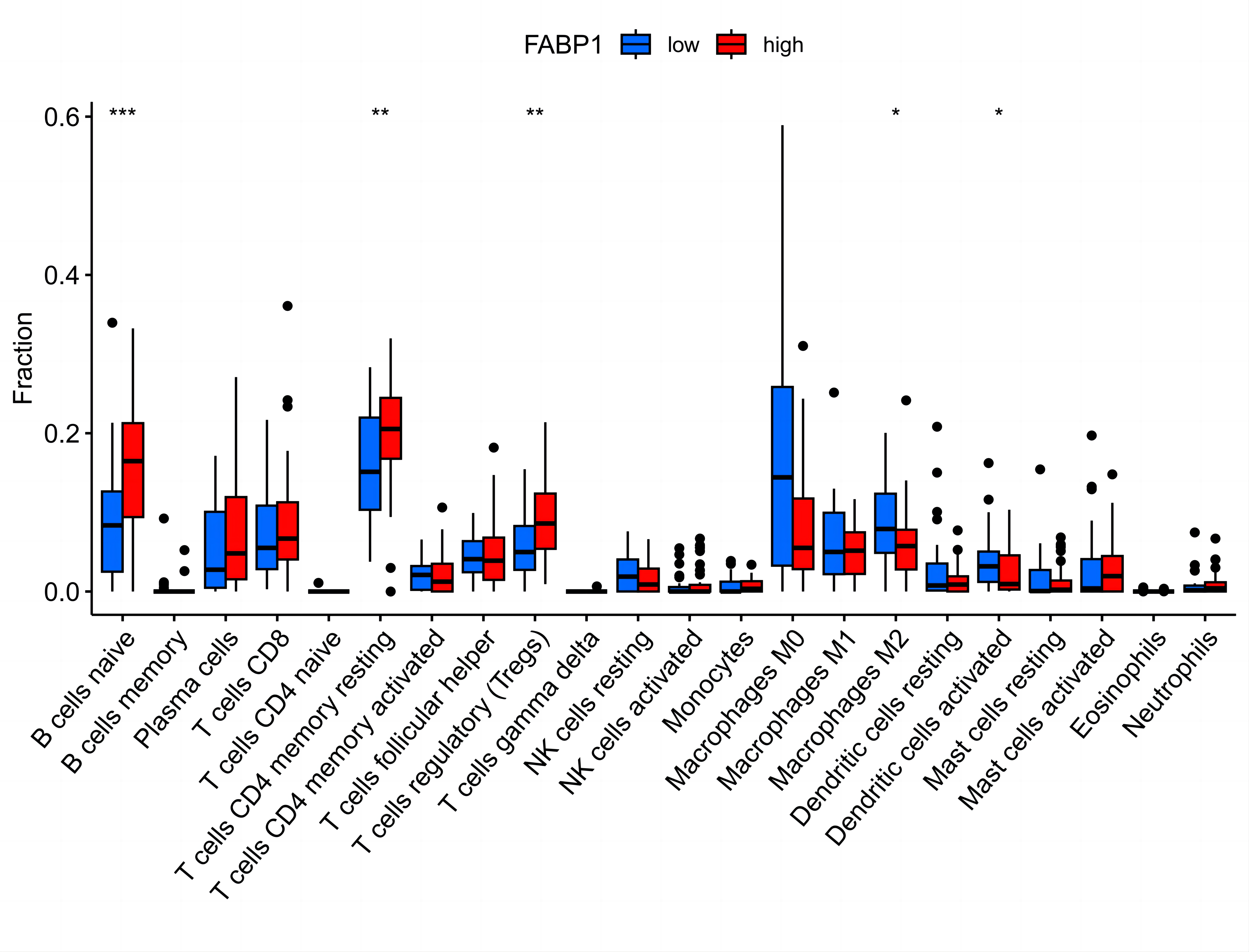

Supplement: Supplementary file 5 [file Image_5.jpeg]
