# Supplementary material for: Prognosis signature for predicting the survival and immunotherapy response in esophageal carcinoma based on cellular senescence-related genes
Source: Front Oncol. 2023 Aug 17;13:1203351. doi: 10.3389/fonc.2023.1203351 (PMC10470646; doi:10.3389/fonc.2023.1203351)
Supplement: Supplementary file 6 [file Table_1.doc]

| DEK | Forward | 5′-TGTTAAGAAAGCAGATAGCAGCACC-3′ |
| --- | --- | --- |
|  | Reverse | 5′-ATTAAAGGTTCATCATCTGAACTATCCTC-3′ |
| RUNX1 | Forward | 5′-AGTGGACGGACCCCGAGAGC-3′ |
|  | Reverse | 5′-ACCGCATGGCACTTCGCCTC-3′ |
| SMARCA4 | Forward | 5′-CCCGTGGACTTCAAGAAGATA-3′ |
|  | Reverse | 5′-CGGCAGACACTGTGATCATTT-3′ |
| SREBF1 | Forward | 5′-AATTGGGCCAGGAATTTGAT-3′ |
|  | Reverse | 5′-ATTCTGGTAGCCGTGACACC-3′ |
| TERT | Forward | 5′-AGCTGACGTGGAAGATGAGC-3′ |
|  | Reverse | 5′-ATCAGCCAGTGCAGGAACTT-3′ |
| TOP1 | Forward | 5′-AGAGCCTCCTGGACTTTTCC-3′ |
|  | Reverse | 5′-ACCACACTGTTCCTCTTCAC-3′ |
| GAPDH | Forward | 5′-GCAAAGTGGAGATTGTTGCCAT-3′ |
|  | Reverse | 5′-CCTTGACTGTGCCGTTGAATTT-3′ |
